# Lung bioengineering: physical stimuli and stem/progenitor cell biology interplay towards biofabricating a functional organ

**DOI:** 10.1186/s12931-016-0477-6

**Published:** 2016-11-28

**Authors:** Paula N. Nonaka, Juan J. Uriarte, Noelia Campillo, Vinicius R. Oliveira, Daniel Navajas, Ramon Farré

**Affiliations:** 1 Unitat de Biofísica i Bioenginyeria, Facultat de Medicina, Universitat de Barcelona, Casanova 143, 08036 Barcelona, Spain; 2 CIBER Enfermedades Respiratorias, Madrid, Spain; 3 Institut de Bioenginyeria de Catalunya, Barcelona, Spain; 4 Institut d’Investigacions Biomèdiques August Pi Sunyer, Barcelona, Spain

**Keywords:** Organ bioengineering, Lung scaffolds, Bioreactor, Stem cell, Physical stimuli, Mechanical microenvironment

## Abstract

A current approach to obtain bioengineered lungs as a future alternative for transplantation is based on seeding stem cells on decellularized lung scaffolds. A fundamental question to be solved in this approach is how to drive stem cell differentiation onto the different lung cell phenotypes. Whereas the use of soluble factors as agents to modulate the fate of stem cells was established from an early stage of the research with this type of cells, it took longer to recognize that the physical microenvironment locally sensed by stem cells (e.g. substrate stiffness, 3D architecture, cyclic stretch, shear stress, air-liquid interface, oxygenation gradient) also contributes to their differentiation. The potential role played by physical stimuli would be particularly relevant in lung bioengineering since cells within the organ are physiologically subjected to two main stimuli required to facilitate efficient gas exchange: air ventilation and blood perfusion across the organ. The present review focuses on describing how the cell mechanical microenvironment can modulate stem cell differentiation and how these stimuli could be incorporated into lung bioreactors for optimizing organ bioengineering.

## Background

Significant respiratory diseases such as chronic pulmonary obstruction, emphysema, idiopathic pulmonary fibrosis, primary pulmonary arterial hypertension, interstitial lung disease, cystic fibrosis and α-1-antitrypsin deficiency result in irreversible structural lung damage, with lung transplantation as the only therapeutic indication when the disease reaches an advanced progression [1]. Unfortunately, the success of lung transplantation is limited because the paucity of viable organs from donors and the incidence of obliterative bronchiolitis resulting from an alloimmune response caused by disparities between donor and recipient human antigens. Indeed, the average survival rate of patients after lung transplantation is currently confined to a few years [1, 2]. Hence, strategies to increase the availability of suitable lungs for transplantation are required, particularly taking into account that the number of potential recipients is increasing due to the progressive aging of the population, adding more potential patients with severe respiratory diseases to the waiting lists for lung transplantation. In this context, one approach currently used is to improve the techniques of ex-vivo lung perfusion for increasing the number of donor lungs that are acceptable for transplantation. Another more ambitious approach regarded as a potential future therapeutic alternative is lung bioengineering, but current research is at early stage and intensive scientific efforts are thus required [3].

This short review aims to address the topic of lung bioengineering, specifically focusing on the potential differentiation effects of the mechanical stimuli sensed by stem cells seeded in decellularized organ scaffolds. The first section briefly refers to acellular lung scaffolds as the currently preferred platform for organ bioengineering. The second section is succinctly devoted to mention that stem/progenitor cells are the ones used to repopulate the acellular lung scaffolds for lung regeneration. Detailed information on stem cells for this application can be found in a very recent review published by this journal [4]. The third and main section is devoted to explain the available information on the effects of mechanical stimuli on stem cell differentiation. The final section briefly explains how different approaches are used to apply mechanical stimuli to recellularized lung scaffolds by means of bioreactors.

## Review

### The organ scaffold as a substrate for lung bioengineering

Some potential approaches for engineering functional tissue elements are based on constructs at the microscale level, for instance as described in lung-on-a-chip applications mimicking the alveolar-capillary membrane [5], or on synthetic scaffolds aimed at reproducing the lung extracellular matrix [6, 7]. However, given the considerable structural complexity of whole lungs, the current approach aimed at organ engineering is mainly based on using the lung’s natural extracellular matrix (ECM) as the starting scaffold for rebuilding the organ [3]. The decellularized lung ECM can maintain the native three-dimensional (3-D) architecture, its biochemical composition and the original microvasculature structure of the organ. These unique properties make this natural scaffold very promising for the bioartificial fabrication of functional lungs since it provides a better recreation of the cellular in vivo microenvironment. Several decellularization techniques have already been used in a diversity of tissue including skin, esophagus, artery, bladder, trachea, liver and heart [8, 9].

Compared to other organs, the structure of the lung is particularly complex, and hence lung decellularization is a complex process. Indeed, the lung is structured as an asymmetrically branching airway tree starting at the trachea and ending at ~300 millions of alveoli. Moreover, there is a whole pulmonary vascular circuit providing blood perfusion to each individual alveolus. These two fluid circuits (for air and blood) are anatomically and functionally matched to facilitate gas exchange through the alveolar-capillary membrane. In addition to its structural complexity, the lung compartments contain up to 60 different types of cells [10]. Despite these difficulties, it has been shown that by using adequate protocols the lung can be decellularized to obtain an almost intact extracellular scaffold [11–20].

In the last few years, some studies have provided proof of concept for lung bioengineering. Price et al. [11] reported that decellularized lung scaffold can be recellularized with pulmonary fetal cells and subjected to simulated ventilation. In addition, Ott et al. [13] and Petersen et al. [14] recellularized rodent lung scaffolds with lung cells to obtain bioengineered lungs, showing limited short-term functionality of gas exchange after in vivo implantation. Although these works represented a milestone in the birth of lung bioengineering, any potential future routine application following the approach of these authors could be hampered by the problem of using differentiated cells, given their limited availability and proliferative capacity [15].

### Potential use of stem cells for lung engineering

Using undifferentiated cells for seeding a lung extracellular matrix could be a more practical strategy for lung bioengineering given the possibility of expanding these cells and their capacity to differentiate into different phenotypes. However, this approach requires that stem cells differentiate into the required lung phenotypes at each specific site within the organ structure.

Interestingly, Cortiella et al. [12] reported that murine embryonic stem cells (mESC) seeded in decellularized lungs secreted extracellular matrix components during their differentiation that were absent in acellular lungs. Moreover, they also reported site-specific stem cell differentiation. Cells into the decellularized trachea and bronchi surface expressed epithelial cell markers and a few of them even presented ciliated phenotypes. In addition, embryonic stem cell (ESC) differentiation towards the endothelial lineage, including formation of very simple capillary-like networks, was also reported. These data strongly suggested that the decellularized lung retains enough scaffold-mediated biological signals to drive stem cells toward lung-specific lineages thereby guiding tissue development in vitro. More recently, Daly et al. [16] cultured acellular lungs with intratracheally inoculated bone marrow-derived mesenchymal stem cells (MSC) and observed how cells attached, migrated, proliferated and transiently expressed lung precursor markers in different regions rich in fibronectin, collagen types I and IV and laminin. However, these previous attempts to bioengineer a lung with stem cells were carried out without providing physical stimuli associated with the main physiological processes in the lung: ventilation and perfusion.

In a study carried out on bioengineered rat lung lobes seeded with human adipose tissue-derived MSC (hAT-MSC) and human bone marrow-derived MSC (hBM-MSC) the authors observed that both cell types could adhere to the decellularized matrix [17]. After culturing lungs in a ventilation/perfusion bioreactor, hBM-MSC and hAT-MSC gave rise to pro-surfactant protein C (pro-SPC) cells. Moreover, hAT-MSC were capable to better adhere to airways and gave rise to Club-like cells, thereby suggesting them as more suitable candidates for lung tissue engineering. Thus, the results presented in that report emphasized the importance of studying different sources of stem cells and their distinct potential to differentiate according to their environment [17].

Other studies performed in rodent [18] and human lungs [19] also showed that the integrity of extracellular matrix plays a key role in cell engraftment. A control acellular lung provides a better cell-adhesion substrate when compared to an emphysematous acellular lung in a 3-D culture. This behavior was abolished when normal decellularized lungs were solubilized and used for cell culture substrate coating, suggesting that the maintenance of the native structure of ECM proteins is important for cell engraftment, and also that a non-defective lung structure could be required for optimized whole organ regeneration [18, 19].

Therefore, the research experience currently available using undifferentiated cells for lung bioengineering indicates that the decellularized organ scaffold would play a fundamental role in modulating important stem cell processes of homing and differentiation, and specially in modulating the secretion of required ECM components which would contribute to tissue reconstruction [20].

### Physical stimuli modulate stem cell differentiation

Stem cells, given their potential to expand in vitro and differentiate onto multiple lineages, are an important target in studying organ regeneration. It is currently well recognized that stem cells multi-potency is modulated by signals of the surrounding local microenvironment, including biophysical and biochemical cues presented by the matrix and soluble mediators secreted by neighbor cells or coming from systemic pathways [12, 21]. In fact, as different organs/tissues are subjected to particular biophysical stimuli during normal function, it is reasonable to expect that physiological conditions could promote stem cell differentiation towards specific phenotypes in vitro. Indeed, physical stimuli have been shown to modulate stem cell differentiation. For instance, electrical stimuli in case of cells directed to the nervous, cardiac and muscular systems, or compression and extension in cells aimed to bioengineer tendons and bones [22–26].

**Fig. 1:**
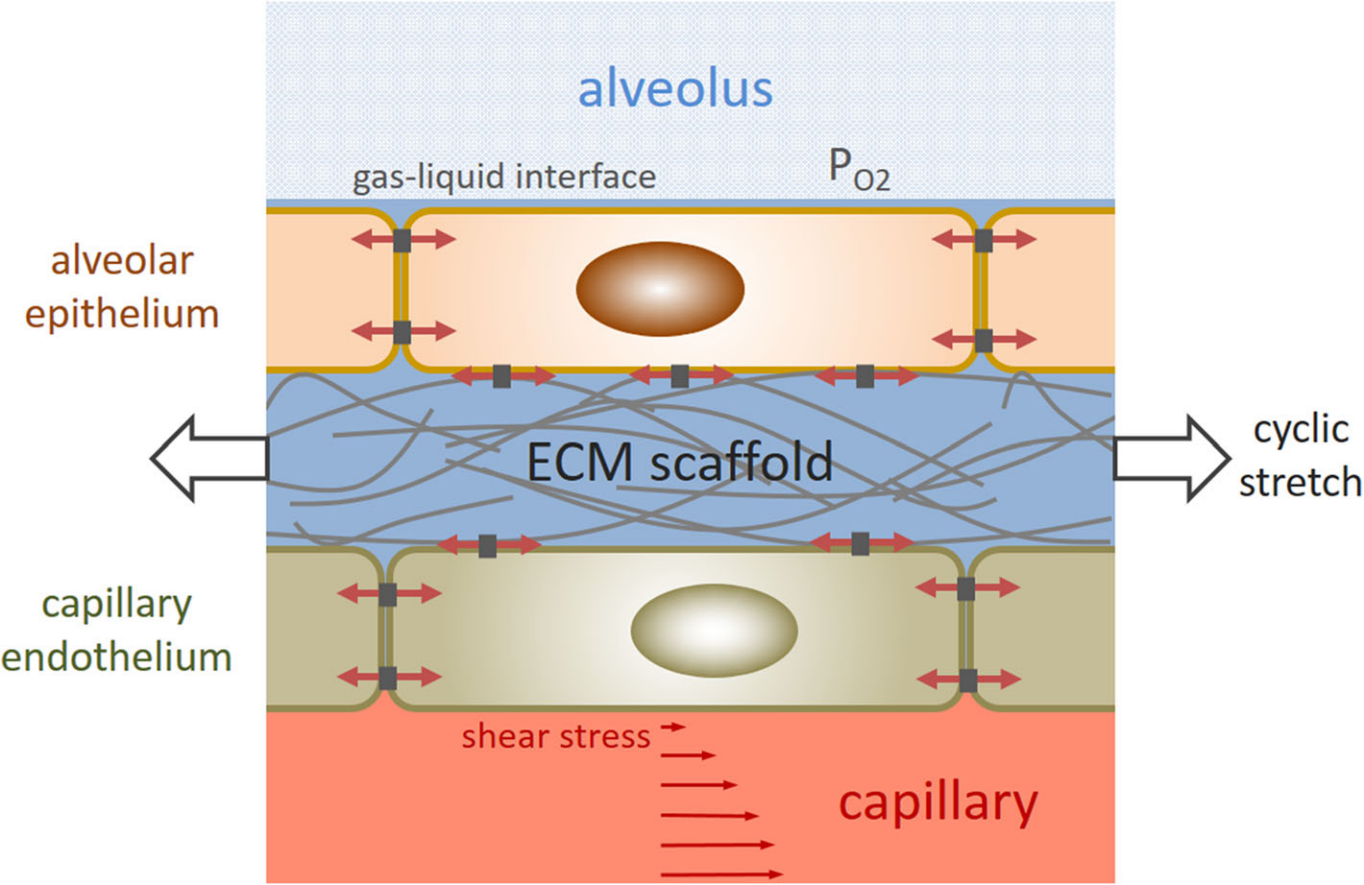
Diagram of the alveolar-capillary membrane illustrating the physical stimuli experienced by cells at physiological conditions of normal breathing. In both alveolar epithelial cells and capillary endothelial cells, cyclic stretch caused by inspiration/expiration changes cell size and modifies the forces (*represented by red arrows*) acting on the cytoskeleton through cell-cell junctions and focal adhesions to the extracellular matrix (ECM) scaffold. Capillary endothelial cells are also subjected to shear stress forces caused by blood circulation. The stiffness of the ECM is sensed by the cytoskeleton through physical contacts in focal adhesions. Oxygen concentration experienced by cells changes along the airways tree and the capillary system. Alveolar epithelial cells are subjected to an air-liquid interface environment. See text for detailed explanation of the effects of these physical stimuli on stem cells

In the case of lung cells (Fig. 1), the most notable mechanical stimuli experienced are: a) substrate microenvironment with varying viscoelasticity and 3-D structure on the different lung sites; b) cyclic stretch caused by the continuous change in lung volume associated to breathing; c) shear-stress due to fluid circulation through airways and vessels; d) existence of an air-liquid interface in the airway epithelium and e) variations in oxygen partial pressure in the various sections of the gas-exchange architecture (e.g. upper and lower airways, alveoli, arterial and venous sides of the capillary). These physical stimuli, when applied alone or in combination with pro-differentiating soluble factors have been shown to modulate in vitro stem cell commitment in both embryonic and adult mesenchymal stem cells, as detailed in the following paragraphs.

Substrate physical properties such as stiffness, 3-D structure and viscoelasticity modulate the differentiation of stem cells. It is well accepted that some aspects of the dynamic 3-D cell environment play an important role in unlocking the full potential of stem cells associated with their renewal, differentiation, and assembly which is observed in native tissues [27–29]. Cells sensing matrix stiffness variation experience cytoskeleton rearrangement with consequent alteration of intracellular tension, changes in morphology and focal adhesion reorganization [30]. Indeed, the stiffness of the substrate in which stem cells are cultured can control their commitment *per se*. For instance, soft matrices that mimic brain stiffness are neurogenic, while stiffer matrices mimicking muscle are myogenic and more rigid matrices that mimic bone are osteogenic [31]. Moreover, cardiomyocyte development also depends on substrate stiffness [32]. Forces applied over the cell junctions may establish distinct effects in stem cell behavior. According to Chowdhury et al. [33], homogeneous cell culture on soft substrates facilitates low cell-matrix traction forces inducing self-renewal and pluripotency [33]. Additionally, Cortiella et al. mention the importance of both substrate properties [34] and 3-D structure [12] for lung bioengineering. How the mechanical properties of the substrate modulate stem cell fate is currently subjected to intense research focusing on both, substrate stiffness and 3-D architecture [35–39].

Cyclic stretch -which can be applied at different frequencies to mimic respiratory rates [40–42]- is a stimulus that considerably affects MSC proliferation and differentiation into different lineages. Biaxial stretch promotes differentiation toward chondrogenic lineage [43], while uniaxial stretch is usually associated with commitment to myogenic lineage [44]. Stretch promotes MSC proliferation when cells are subjected to elevated strain amplitude and high frequency rates, upregulating smooth muscle α-actin, reorientating actin fibers which lead to differentiation into smooth muscle cell phenotype [45]. The differentiating effects of cyclic stretch depend, however, on whether the protocol is conducted in 2-D or 3-D microenvironments [46]. Spontaneous breathing cycles, causing rhythmic inflation/deflation of lungs, exert mechanical forces which provide cues that modulate cell growth, survival and direct stem cells fate [21]. Moreover, distinct mechanical environments such as compressive and tensile strain provoke different structural distortions and change cell volumes, in a way that influence gene expression and morphology [30, 47]. Recent published data provide further insight into the role played by cyclic stretch in modulating stem cell differentiation [48–51].

Shear stress is of particular interest in cells of the blood vessels. It is known that this mechanical stimulus modulates endothelial function and, potentially, endothelial specification of stem cells [21, 52]. Wang et al. [53] demonstrated that laminar shear stress promotes endothelial cell fate in a murine mesenchymal progenitor cell line: cells aligned in the direction of flow, upregulated endothelial cell markers and formed capillary-like tube structures. The increased mRNA levels of VEGF found in cells exposed to shear stress gives support to the notion that this stimulus could regulate the expression of growth factors, creating a positive feedback loop promoting this specific cell fate. Moreover, human cord blood endothelial progenitor cells co-cultured with vascular smooth muscle cells increased their endothelial differentiation potential when exposed to laminar shear stress [54]. Complementary studies in this field are contributing to our understanding of the role played by continuous and cyclic shear stress in stem cell modulation [49, 55–57].

The existence of an air/liquid interface is another factor that can considerably affect stem cell fate into lung phenotypes [58, 59]. Van Haute et al. [59] described a protocol for obtaining lung epithelial-like tissue from human embryonic stem cells using an air-liquid interface system to mimic airway conditions. Petersen et al. [14] also observed that ventilation with air increased type I alveolar epithelial cells as well as the number of ciliated columnar epithelial cells. The air-liquid interface setting is progressively being used to optimize the differentiation of stem cells into airway and alveolar lung epithelial phenotypes [60–63].

The role played by oxygen partial pressure in stem cell differentiation has been scarcely examined and requires more detailed work [64–66]. Indeed, whereas most of the research has been carried out under room-air oxygenation conditions (~21% O_2_), it is well documented that, in vivo, both embryonic and adult tissues, and specifically the different lung compartments, are subjected to oxygen partial pressures far below the ones corresponding to room air [67]. Given that embryogenesis is heavily influenced by oxygen gradients, small shifts in oxygen tension have shown to stimulate differentiation into many cell types: dopaminergic neurons [68], cardiomyocytes [69], chondrocytes [70], and, most particularly, endothelial cells [71]. More specifically, adult adipose [72] and murine bone-marrow-derived [73] MSC have shown different differentiation fates depending on oxygen concentration. A more recent study has demonstrated that ESC and induced-pluripotent stem cells (iPSC) cultured in a 5% oxygen concentration present enhanced formation of embryonic bodies with increased capacity to differentiate into mature lung epithelial cell phenotypes, being iPSC more affected by oxygen gradient variation [74]. This positive role of using oxygen physiological values in vitro to promote stem cell differentiation towards lung phenotypes has been also recently confirmed [75].

Therefore, the existing experimental evidence above mentioned gives support to the concept that the physical microenvironment significantly contributes to modulation of the differentiation of both embryonic and adult stem cells. However, specific data concerning stem cell differentiation towards lung phenotypes by physical stimuli are scarce. In an interesting study, decellularized rat lungs were recellularized with human iPSCs-derived endothelial cells and iPSCs-derived epithelial progenitor cells via pulmonary artery and trachea, respectively. The whole lung was then cultured for 12–15 days under vascular perfusion and the results showed mature lungs phenotypes expressed by Nkx2.1, CD31, T1α/podoplanin and CC10 markers [76]. In another study, iPSC-derived alveolar epithelial cells type II cultured into acellular rat lungs were subjected to vascular perfusion and the authors were able to diffusely repopulate alveolar lung structures and also observed epithelial lung cell markers. Nevertheless, these results were obtained under conditions deprived of the biophysical stimulus corresponding to lung ventilation [77].

It is of note that most reported studies focus on the effect of an individual physical stimulus on stem cell fate. However, cells differentiating in vivo are subjected to a variety of simultaneous stimuli: a myriad of physiological soluble factors and specific molecular composition of the extracellular matrix in addition to different general physical factors such as 3-D architecture and substrate stiffness. In the case of cell lungs, additional mechanical stimuli are active: cyclic stretch, shear stress, air-liquid interface or oxygen gradients. Although there is not much information on the combined effects of these many stimuli on stem cell differentiation, it seems reasonable to expect different potential outcomes: from counterbalancing the effect of each other to additive or synergistic responses. In fact, the relatively scarce information available suggests that the result would depend on what specific mechanical stimuli are combined and on their relative intensity [78–81]. Owing to this lack of data, comparing the results on stem cell differentiation obtained from very simple experiments involving only one or two mechanical stimuli in vitro with the actual differentiation experienced by endogenous adult stem/progenitor cells in the in vivo lung is currently difficult.

It is worth noting that a considerable number of studies focusing on how mechanical stimuli modulate the fate of stem cells have been carried out on MSC, probably because, as compared with other stem cells, they are easily obtained from patients even for autologous applications. While these works are of great interest to improve our understanding of the mechanisms involved in stem cell mechanotransduction, their impact on the specific field of lung bioengineering is probably limited since there are no evidences that MSC can differentiate into different types of lung cells. In fact, it has been suggested that the role of these adult stem cells in lung bioengineering could be to remodel the extracellular matrix of the scaffold for providing a better microenvironment to other infused cells capable of differentiation into lung phenotypes [82].

### Biophysical stimuli in bioreactors for lung bioengineering

Given the potential effects induced by physical stimuli on stem cells within the lung, it is expected that optimizing the bioengineering process would require a bioreactor capable of applying ventilation, perfusion and oxygenation conditions facilitating migration, cell-ECM adhesion, proliferation and differentiation of the stem cells seeded into an acellular lung scaffold.

Lung bioreactors should include general characteristics of other organ bioreactors (e.g. temperature control, media perfusion, isolation and sterile conditions), much of them in common with conventional settings to maintain lungs under *ex vivo* conditions [83]. Moreover, the bioreactor should be equipped with sensors (e.g. flow, volume, pressure) to allow monitoring the most important physiological variables. A control system, preferably in closed-loop mode, should be able to adapt perfusion and ventilation to potential changes in the mechanical properties of the airway and vascular compartments [84, 85].

Lung bioengineering studies performed in the last years have described a variety of methods and protocols for cell seeding and culturing into a lung scaffold, making it difficult to compare the reported results [12–14]. These studies started with mouse and rat models and employed bioreactors based on methodologies such as diffusion [12], dynamic rotating wall vessel [86], airways ventilation [11] or both airway ventilation and vascular perfusion [13, 14]. In one of the first works [13], a rodent acellular lung was recellularized and subjected to liquid ventilation followed by air ventilation, both positive-pressure controlled and with continuous vascular perfusion. The authors observed that seeding lungs with human umbilical cord endothelial cells (HUVECs) and rat fetal lung cells (FLCs) resulted in closely physiological ventilation and reestablishment of an alveolar-capillary barrier and gas exchange. Another early study performed using only liquid negative-pressure ventilation on scaffold-seeded neonatal lung epithelial cells showed similar results [14]. Employing the same bioreactor model, Mendez et al. [17] cultivated rat lung scaffolds with human MSC and observed the capacity of these cells to differentiate into epithelial cells. Interestingly, Wagner et al. [87] developed an alternative model to study site-specific cell-matrix interactions, consisting in seeding cells in small pieces of human lungs and inoculated the airways with human lung fibroblasts, human bronchial epithelial cells or human bone marrow-derived MSC and blood vessels with human vascular endothelial cells. The authors reported that cells survived for at least 28 days.

Bonvillain et al. [82] adapted the usual system for small rodents to a large organ bioreactor and performed a study in macaque lungs, seeding the scaffold with macaque bone marrow-derived MSC or lung-derived microvascular endothelial cells and observed that MSC lined the alveolar septa. The authors reported a good efficiency in inoculating distal lung tissue: large airways presented a monolayer of squamous-like MSC after 14 days of culture in negative-pressure ventilation. The authors also found cells lining the small vasculature under constant vascular perfusion. Despite this study contributed to our understanding of cell-matrix interactions in acellular lungs, the authors did not achieve complete recellularization. A clinical-scale bioreactor allowing an isolated lung culture (porcine and human scale) with oscillatory perfusion through the pulmonary artery and negative pressure ventilation was developed by Charest et al. [84]. Using this bioreactor, the organ under biofabrication experienced mechanical stimuli similar to the physiological ones when in vivo lung ventilation was driven by the negative pressure caused by thoracic cage expansion. Interestingly, negative pressure ventilation seems to enhance survival and secretion clearance of epithelium in small airways resulting in a more recruited/oxygenated lung and reduced lung injury [14, 88]. However, it is still not clear whether positive or negative pressure ventilation results in significant differences [89]. Some recent studies with large size organs have been performed by using commercial bioreactors [90]. Nichols et al. [91] decellularized porcine and human lungs using a large bioreactor and obtained suitable scaffolds for regeneration. Seeded cells –such as murine embryonic stem cells, human fetal lung cells, bone marrow derived mesenchymal stem cells and human alveolar epithelial cells– presented good adherence, viability and reduced immunogenicity when compared to the ones seeded in synthetic matrices.

A remarkable study by Ren et al. [92] focused on the specific problem of vascular endothelization in lung scaffolds. These authors infused acellular lungs with human cells, including endothelial and perivascular cells derived from induced pluripotent stem cells, using a two-step protocol and achieved a significant level of vascular endothelization. Interestingly, the vascular resistance and barrier function of the new endothelium were optimized in vitro and 3-day after transplantation in rats the vessels remained patent. Another relevant study recently published describes human lung recellularization in a bioreactor culture and compares distinct methods of obtaining and seeding cells in adult and pediatric lungs [15]. The authors succeeded in repopulating pediatric lung scaffolds using primary lung-derived vascular, epithelial and tracheal/bronchial cells, resulting in bioengineered lungs with the presence of type I and II alveolar epithelial cells along the whole organ, alveolar-capillary junctions and increased collagen I content when compared to the decellularized scaffold. These lungs also presented normal values of static compliance. Two important elements that must be highlighted on the bioreactor culture methodology used in that work are the pre-treatment of the scaffolds with human platelet-rich plasma and the slow flow rate during cell inoculation, which seemed to yield an improved cell attachment and consequently better vascular and lung tissue formation. More recently, a novel bioreactor approach that could facilitate translational progress in lung bioengineering was described by Raredon et al. [93]. The system is based in the simulation of the sealed lung compartment by a silicone pleural membrane to apply ventilation by negative pressure.

Improving our understanding of the mechanobiology of stem cells should help in optimizing the ventilation and perfusion settings of the bioreactor for lung bioengineering. Indeed, better understanding the effect of the air-liquid interface on cell homing and differentiation could provide clues on selecting air or liquid ventilation at the different stages of lung biofabrication. Moreover, insight into the role played by compression in different cell types may suggest whether negative pleural pressure ventilation is preferable to positive airway pressure ventilation. Furthermore, better understanding how static and dynamic stretch modulate stem cell fate should be useful for determining the optimal frequencies and amplitudes of ventilation, the most suitable lung inflation volume at end expiration and whether continuous or pulsatile perfusion is more adequate.

## Conclusions

Given the role that physical stimuli play in stem cell fate, using optimized bioreactors providing stimuli adapted to lung physiology (ventilation and perfusion) is expected to be crucial for the progress of lung bioengineering. Current works in this field of regenerative medicine go beyond research using lung slices and are carried out in whole organs within complex bioreactors, thus providing useful information and anticipating more scientific progress. However, although it has considerably progressed in the last years, the field of lung bioengineering is still in its infancy. Accordingly, the information available is scarce and difficult to interpret, in particular because some findings have been reported by just one or few laboratories and hence ample reproducibility is still lacking. Also, some methodologies should be improved and more widely used, for instance those characterizing the composition and immunogenicity of decellularized lungs and those adequately identifying the phenotype of differentiated cells. More generally, there are still key questions that need to be clarified and much better understood. Specifically, which are the most suitable cell types, media and growth factors (being probably different for the airway and vascular compartments), and how to provide the optimal conditions of ventilation, perfusion and oxygenation to the lung along the process of biofabrication. Advancing into the solution of these open issues requires interdisciplinary research joining stem cell biologists, lung physiologists and experts in biophysics and tissue engineering methodologies.
